# Wound Healing Problems in the Mouth

**DOI:** 10.3389/fphys.2016.00507

**Published:** 2016-11-02

**Authors:** Constantinus Politis, Joseph Schoenaers, Reinhilde Jacobs, Jimoh O. Agbaje

**Affiliations:** ^1^OMFS-IMPATH Research Group, Department of Imaging and Pathology, Katholieke Universiteit LeuvenLeuven, Belgium; ^2^Oral and Maxillofacial Surgery, Leuven University HospitalsLeuven, Belgium

**Keywords:** oral, wound healing, dental implant, nerve damage, local and general factors, inflammation, granulation tissue

## Abstract

Wound healing is a primary survival mechanism that is largely taken for granted. The literature includes relatively little information about disturbed wound healing, and there is no acceptable classification describing wound healing process in the oral region. Wound healing comprises a sequence of complex biological processes. All tissues follow an essentially identical pattern to complete the healing process with minimal scar formation. The oral cavity is a remarkable environment in which wound healing occurs in warm oral fluid containing millions of microorganisms. The present review provides a basic overview of the wound healing process and with a discussion of the local and general factors that play roles in achieving efficient would healing. Results of oral cavity wound healing can vary from a clinically healed wound without scar formation and with histologically normal connective tissue under epithelial cells to extreme forms of trismus caused by fibrosis. Many local and general factors affect oral wound healing, and an improved understanding of these factors will help to address issues that lead to poor oral wound healing.

## Introduction

In Greek mythology, the Rod of Asclepius is a serpent-entwined rod wielded by the Greek god Asclepius. Wounded patients could be healed if they were brought to the temple and the serpent licked their wounds during the night (Gardner, 1925). The Rod of Asclepius is still used as a symbol associated with modern medicine and health care. Wound healing is a primary survival mechanism that is largely taken for granted. Although, wound healing has long been considered a primary aspect of medical practice, disturbed wound healing is infrequently discussed in the literature, and there is no acceptable classification to describe wound healing processes in the oral region.

Wound healing entails a sequence of complex biological processes (Bielefeld et al., 2013). All tissues follow an essentially identical pattern to promote healing with minimal scar formation. One fundamental difference between would healing and regeneration is that all tissues are capable of renewal, but healed tissue does not always possess the same functionality or morphology as the lost tissue (Takeo et al., 2015). Moreover, wound healing is a protective function of the body that focuses on quick recovery (Wong et al., 2013), whereas the process of regeneration in an hostile environment takes more time. In particular, the oral cavity is a remarkable environment in which wound healing occurs in warm oral fluid that contains millions of microorganisms.

The present review provides a basic overview of wound healing, focusing on specific characteristics of the process of wound healing in the oral cavity. We also discuss local and general factors that play roles in achieving efficient wound healing.

## Normal wound healing process

Wound healing requires multiple finely tuned processes that occur in a specific sequence (Bielefeld et al., 2013; Eming et al., 2014). Intact hemostatic and inflammatory mechanisms are needed, and mesenchymal cells must migrate to the wounded area and proliferate at site of injury. To maintain organization and give rise to newly formed tissue, angiogenesis and epithelialization occur, together with collagen synthesis, binding, and alignment which causing open wounds to contract (Bielefeld et al., 2013; Eming et al., 2014; Takeo et al., 2015). Further wound healing involves regeneration (the substitution of damaged tissues with the same cell type) and fibrosis (replacement of damaged tissues with connective tissue). These processes involve three types of cells. First, epithelial cells are required, which can continuously regenerate. For the secondary stage of organization, there is also a need for cells that replicate on a lower level but that can replicate at a high rate in the presence of a stimuli to recover the original tissue—for example, fibroblasts and vascular endothelial cells. Finally, cells that lack the ability to divide are required, such as cells of the peripheral nerve system and odontoblasts (Mahdavian et al., 2011; Reinke and Sorg, 2012; Bielefeld et al., 2013; Wong et al., 2013).

## Wound healing process sequence

Wound healing is initiated by the formation of a blood clot that closes the wound. Vasoconstriction occurs to stop the bleeding, followed by platelet activation (Cooper, 1999; Broughton et al., 2006). Platelets play several important roles within a wound, including regulating primary hemostasis during the aggregation phase and secondary hemostasis during the coagulation phase. Platelets produce biologically active products, including vasoactive mediators and chemotactic factors, such as proteases, cytokines, and growth factors (Broughton et al., 2006; Cooper, 1999). Cytokines send out chemotactic signals to inflammatory cells and local cell populations. The fibrin–fibronectin clot formed during secondary hemostasis serves as a temporary matrix to allow epithelial cells and fibroblasts to migrate into the wound. Upon clot formation, thrombin is activated to prevent excessive blood clotting (Cooper, 1999; Diegelmann and Evans, 2004; Broughton et al., 2006). During the tertiary hemostatic phase, fibrinolytic system activation leads to fibrin degradation. The peptides released during this process provoke chemotaxis and increase capillary permeability (Cooper, 1999; Diegelmann and Evans, 2004; Broughton et al., 2006).

Cytokines initiate an inflammatory reaction that serves to remove debris, damaged or necrotic tissues, and microorganisms (Gharaee-Kermani and Phan, 2001; Li et al., 2007). Blood vessels situated near the wound show dilatation, increased capillary permeability with plasma exudation, and decreased blood flow (Cohen et al., 1992; Li et al., 2007). Leucocytes are attracted to the center of the wound, which quickly becomes saturated with granulocytes and macrophages. In the early stage, primary neutrophilic granulocytes are involved in protecting against bacterial invasion, while macrophages become the dominant cells after a few days (Cohen et al., 1992; Mutschler, 2012). Macrophages also play major roles in initiating collagen synthesis, and in the formation of endothelial cells and fibroblasts. Overall, macrophages act as the engine of the wound healing process (Cohen et al., 1992; Mutschler, 2012). Granulocytes and macrophages both exhibit an anaerobic metabolism, produce collagenase, feed on bacterial debris, and produce lactate, which decreases the tissue pH (Cohen et al., 1992; Velnar et al., 2009). Like hemostasis, this process also must be regulated. Acceleration, amplification, and downregulation are important mechanisms in the normal wound healing process. Prostaglandins and leukotrienes are pro-inflammatory mediators, while inflammatory reactions are suppressed by substances such as lipoxins, resolvins, and protectins (Cohen et al., 1992; Gharaee-Kermani and Phan, 2001; Velnar et al., 2009; Ozturk and Ermertcan, 2011).

This inflammatory phase is followed by granulation tissue formation, re-epithelialization, and formation of a connective tissue matrix (Broughton et al., 2006; Rodriguez-Merchan, 2012). Granulation tissue comprises a dense population of macrophages, fibroblasts, capillary networks, fibronectin, hyaluronic acid and endothelial cells (Rodriguez-Merchan, 2012; Discepoli et al., 2013). Macrophages, fibroblasts, and endothelial cells are interdependent during granulation tissue formation (Rodriguez-Merchan, 2012; Discepoli et al., 2013). Hypoxia is an important trigger for neovascularization during this phase (Schreml et al., 2010; Larjava, 2012). Fluid continues to leak from the wound until basal membrane formation (Kirsner and Eaglstein, 1993; Broughton et al., 2006). At this time, fibroblasts are recruited from the wound edges, and circulating fibrocytes and mesenchymal progenitor cells migrate to the immature connective tissue matrix (Kirsner and Eaglstein, 1993; Cooper, 1999; Broughton et al., 2006).

Re-epithelialization starts from the wound edge, where epithelial cells lose their hemi-desmosomal connections and migrate through the provisional fibrin–fibronectin matrix through the wound until they encounter identical cells (Kirsner and Eaglstein, 1993; Cooper, 1999; Broughton et al., 2006; Li et al., 2007). Targeted migration and proliferation through a loose underlying network requires an efficient, balanced, and enzymatically supported procedure of “cutting and pasting” (Hunt et al., 2000; Broughton et al., 2006; Li et al., 2007; Ozturk and Ermertcan, 2011). The process through which the epithelial cells of two wound edges make direct contact is called healing by primary intention (Cohen et al., 1992; Vanwijck, 2001; Broughton et al., 2006). On the other hand, healing by secondary intention occurs when migrating cells make a connection after a certain time, through granulation tissue or not (Cohen et al., 1992; Vanwijck, 2001; Broughton et al., 2006). The healing process is slower in open wounds due to delayed epithelial closure and a higher rate of granulation tissue formation (Cohen et al., 1992; Vanwijck, 2001; Sorensen, 2012; Glim et al., 2013; Karamanos et al., 2015). The fibronectin connective tissue form is initially loose, with gradual replacement by bigger and stronger collagen bundles. This protects wound against damage from traction and pressure. Extracellular matrix formation is initiated at the wound edge, and gradually progresses to the center/core of the wound. This process is similar to the matrix maturation, as both are initiated at the edge and gradually continue toward the wound center. During this maturation process, new blood vessel formation decreases (Cohen et al., 1992; Broughton et al., 2006; Velnar et al., 2009; Ozturk and Ermertcan, 2011).

The final stage of the wound healing process is called the contraction phase, which begins following sufficient collagen formation in the granular tissue. In the contraction phase, the distance between wound edges is closed, reducing the wound surface, and fastening the wound closure. This last process occurs due to differentiation of fibroblasts and other progenitor cells into myofibroblasts. Myofibroblasts with an actin-enriched cytoskeleton provide matrix constriction. Wound contraction is followed by the remodeling process, in which matrix production stops, fibroblasts are degraded, and myofibroblasts enter apoptosis (Cohen et al., 1992; Broughton et al., 2006; Velnar et al., 2009; Ozturk and Ermertcan, 2011). The final result of wound healing can range from a clinically healed wound with no scar formation and with histologically normal connective tissue under epithelial cells to extreme forms of trismus caused by fibrosis.

### Timing of wound healing phases

Within the wound healing process, the inflammatory phase involves homeostasis and inflammation, which start at the moment of injury and continue for up to 4 to 6 days. The proliferation phase involves epithelialization, angiogenesis, granulation tissue formation, and collagen deposition, and takes place from day 4 to day 14 after injury. Epithelial cell migration starts after 24 h. The maturation and remodeling phase starts from day 8 after injury and proceeds for about a year (Kirsner and Eaglstein, 1993; Ennis and Meneses, 2000; Hunt et al., 2000; Broughton et al., 2006; Larjava, 2012).

## Specific characteristics of the oral cavity wound healing process

### Healing of the palate

Wound healing in the oral cavity is typically characterized by healing of the palate and gingival tissue in the presence of healthy underlying bones and without scar tissue formation (Glim et al., 2013). This is due to early onset of the inflammatory phase, decreased levels of immunity mediators, fewer blood vessels, more cells originating from the bone marrow, rapid re-epithelialization, and rapid fibroblast proliferation (Funato et al., 1999; Glim et al., 2013). In fetal wound healing, the inflammatory phase is absent (Larson et al., 2010).

Wound healing in the palate is more difficult in the absence of healthy underlying bone. In such cases, wound healing might be accompanied by perforation to the nose and antrum or by serious scarring. This can lead to narrowing of the transversal width of the maxilla if the patient is in their growing phase, as is observed in cleft patients who have surgery of the palate (Broughton et al., 2006; Larjava, 2012).

### Periodontal healing

Healing after a tooth extraction follows the same pattern, with the inclusion of a bone healing process (Larjava, 2012; Discepoli et al., 2013). Minutes after the tooth is extracted, the alveoli are closed via blood clotting. Re-epithelialization starts 24 h post-extraction. After 1 week, the blood clot is replaced with granulation tissue. After 8 weeks, the extraction cavity is filled with bone (Larjava, 2012; Discepoli et al., 2013). The bone remodeling process continues for 6 months after the extraction, and is accompanied by a loss of alveolar width and length due to resorption and remodeling (Discepoli et al., 2013). The amount of bone loss varies among individuals, and depends on the location, the presence of adjacent teeth, the treatment protocol, smoking behavior, and the use of membranes and bone substitutes (Trombelli et al., 2008). A review of the effects of plasma concentrates showed reduced post-surgical pain and burden, but no improvement or increase of hard tissue regeneration (Moraschini and Barboza, 2015).

Wound healing after periodontal surgery carries a notable risk of degradation of the interdental papilla, with the occurrence of black triangles. Specific incisions and flaps are important to ensure minimum retraction, and good oral hygiene is critical to avoid inflammation (Larjava, 2012; Discepoli et al., 2013; Glim et al., 2013; Lindhe et al., 2015).

### Healing at dental implant interfaces

A specific form of wound healing occurs around dental implants. In biphasic dental implant procedures, the implant is placed directly under or at the same level as the bone surface. The cover screw is overlaid with soft tissues that heal without substantial granulation tissue formation. Healing in the bone occurs between the edge surface of the implant and the prepared osteotomy edge (Larjava, 2012; Lindhe et al., 2015). Blood clots form mainly at the inner side of the implant grooves, and are then infiltrated by granulocytes and macrophages. Fibroblastic progenitor cells migrate into the provisional matrix, enabling formation of granulation tissue, which is then vascularized by endothelial cell migration. Finally, the cells in the granulation tissue differentiate into osteoblasts, creating bone. This bone formation starts 4 days after placement of dental implants, achieving maximum bone–implant contact after 3 months (Larjava, 2012). Depending on the mechanical stress caused by occlusal forces, bone remodeling around the dental implant persists for at least 1 year.

### Dental pulp healing

Dental pulp healing mainly relies on preservation of the apical blood supply and survival of the damaged odontoblast layer (Goldberg, 2011; Dimitrova-Nakov et al., 2014). If the odontoblast layer is sufficiently intact, the odontoblasts and Hoehl's cells can influence the formation of tubular or atubular reactionary (tertiary) dentin (Goldberg, 2011; Dimitrova-Nakov et al., 2014). Additionally, an opening of the pulp that causes only superficial dental pulp necrosis can provoke an inflammatory reaction that attracts pulpal stem cells or progenitor cells to the wound edge, where they differentiate into odontoblast-like cells that produce reparative dentin or osteodentin. However, persistent inflammation in the dental pulp can lead to pulp necrosis (Goldberg, 2011).

### Bone healing

Jawbone fractures can heal by primary or secondary intention. Bone healing by primary intention (also termed direct bone healing) occurs without callus formation, given the presence of a good blood supply and an existing stable fixation without mobility of the bone fragments—which must be in direct contact with each other with a fracture gap of up to 1 mm (Adell et al., 1987; Discepoli et al., 2013). Under these conditions, osteoblasts are activated within 1 to 3 days, and osteoid is established within a week. Bridging the gap with primary bone can take 4 to 6 weeks depending on the gap distance. This primary bone contains large numbers of osteocytes and collagen fibers intertwined (woven bone) to create a spatial braid or plexus (Adell et al., 1987; Broughton et al., 2006). This bone is subsequently replaced by mature secondary bone that is characterized by a parallel collagen fiber arrangement. The concentric lamellae (lamellar bone) are better calcified, making secondary bone stronger than primary bone (Hom et al., 2009).

Bone healing by secondary intention (also termed indirect bone healing) is characterized by callus formation, and is by far the most important form of bone healing in maxillo-facial surgery (Adell et al., 1987; Broughton et al., 2006). The indirect bone healing sequence can be summarized as follows: (1) inflammation and hematoma formation, (2) fragment stabilization by periosteal and endosteal callus formation, (3) continuity restoration by membranous and endochondral bone formation, and (4) formation of osteons and Haversian canals, and functional adaptation (Junqueira and Carneiro, 2007). After blood clot formation, osteocytes die on both sides of the fracture and the bone matrix degrades. This is followed by the recovery phase in which dead cells, the thrombus, and the degraded bone matrix are cleaned up (Adell et al., 1987; Broughton et al., 2006; Discepoli et al., 2013). The surrounding tissue prompts revascularization and angiogenesis, together with strong cell proliferation. The nature of the formed tissue is associated with the fracture stability and the tissue vascularization. In cases of less stable fracture or poor vascularization, a cartilaginous callus forms first, which is then converted to endochondral bone, followed by further transformation into woven bone, and finally by conversion to secondary (lamellar) bone as the stabilization improves. When bone fragments show high mobility or a large gap, and in cases of periosteum damage, a connective tissue-like callus is formed that clinically appears as a pseudarthrosis in which the bone fragments opposite each other remain mobile (Broughton et al., 2006; Guo and Dipietro, 2010; Discepoli et al., 2013).

### Healing of facial burns

Burns on the face or in the mouth often exhibit compromised wound healing with pronounced scarring (Guo and Dipietro, 2010; Glim et al., 2013). The extent of thermal damage depends on the temperature, contact time, skin or mucosa thickness, and degree of vascularization within the injured area. Due to vascular and inflammatory reactions, burns continue to damage deeper structures for 48–72 h following the initial insult (Larjava, 2012).

### Healing of large defects

Wound healing problems are also commonly associated with reconstructive surgery of congenital defects, trauma, or tumors, as well as preprosthetic reconstructive surgery (Hollander and Singer, 1999; Guo and Dipietro, 2010; Dunda et al., 2015; Karamanos et al., 2015). Substantial morbidity in mouth, head, and neck surgery is influenced by many factors, including existing scars, large defect size, non-vascularized autologous bone grafts or free bone grafts, poor oral hygiene, difficult wound closure, and pre-existing or postoperative infections (Guo and Dipietro, 2010; Dunda et al., 2015; Karamanos et al., 2015).

## Clinical manifestations of disturbed wound healing in the mouth

**Figure 1:**
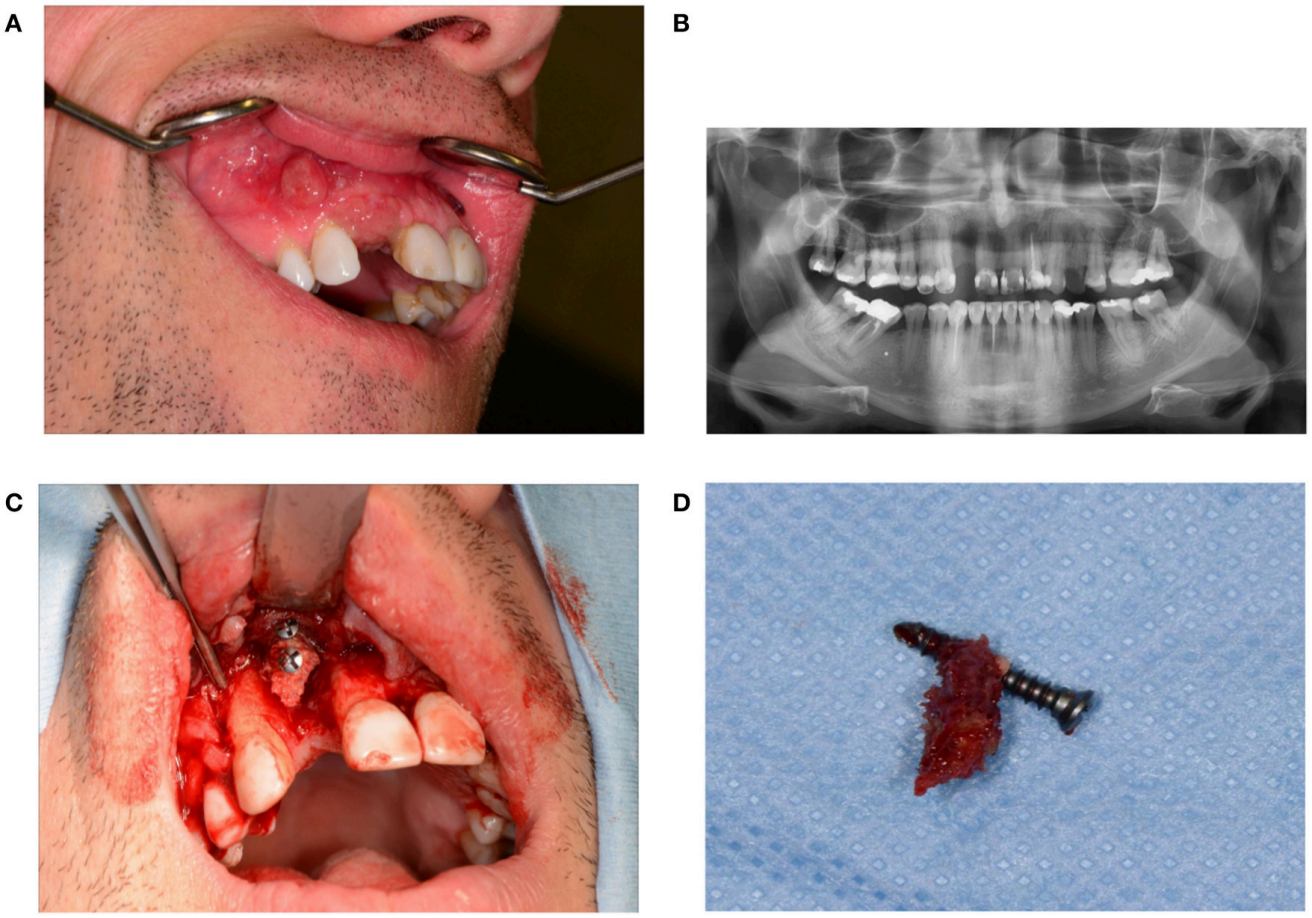
**(A)** Granuloma formation after reconstruction surgery through a bone from the anterior iliac crest in region 12. **(B)** Panoramic radiography indicates a vertical bone deficit in region 12. **(C)** Exploration of a wound from which the residue of a non-healed bone graft was removed. A large part of the originally implanted bone graft has already disappeared due to necrosis and/or resorption. **(D)** The necrotic bone graft with an osteosynthesis screw.

**Figure 2:**
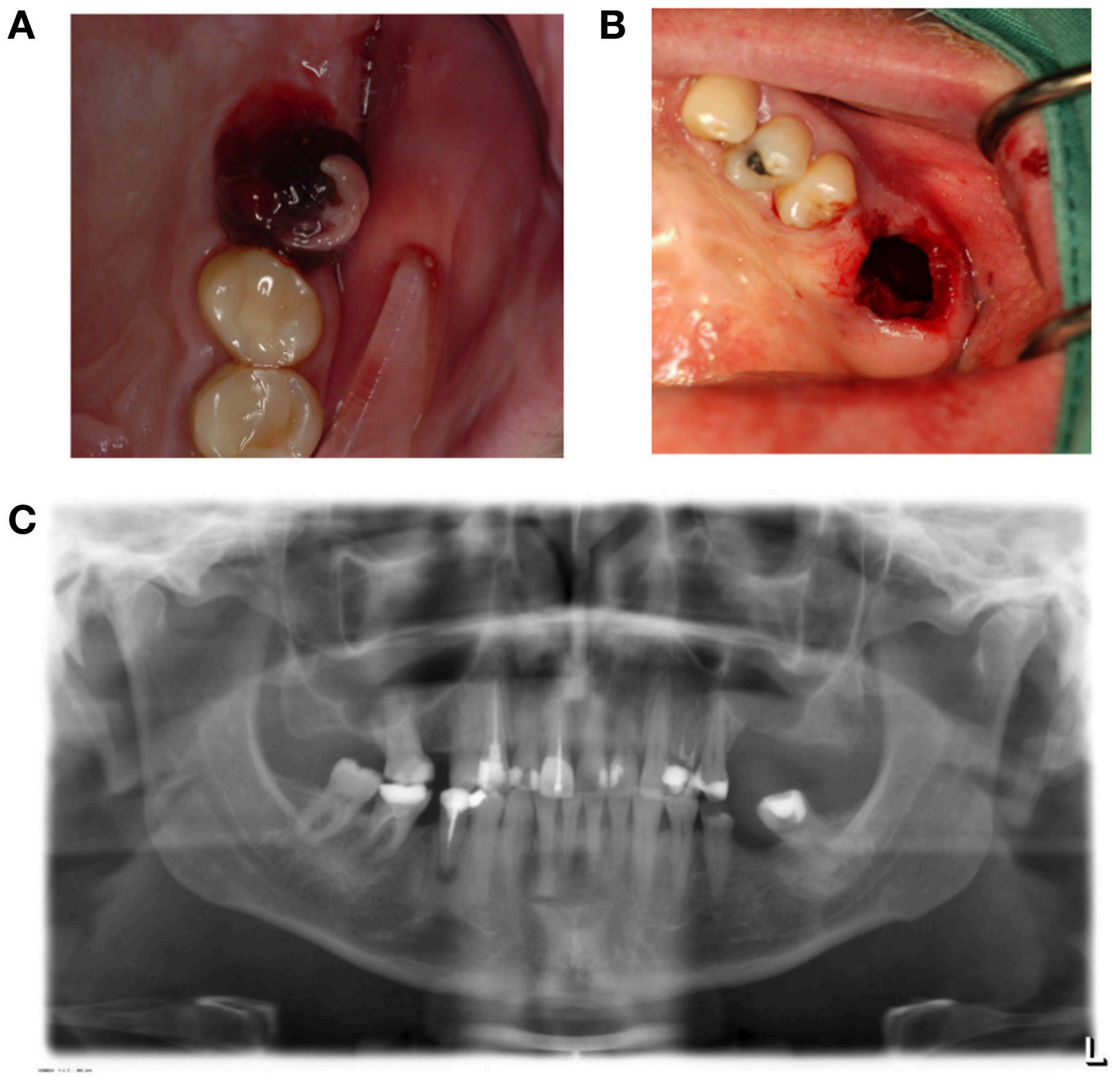
**(A)** A sinus polyp is a manifestation of a poorly healing antrum perforation that does not spontaneously close. This can lead to the bony height of the rest alveolus being too small following tooth extraction, and the connection between the mouth and antrum being too wide. **(B)** Panoramic radiography shows loss of bone height separating the antrum of the oral cavity in region 26. **(C)** Brightening of the wound edges reveals the dimension of the connection between the antrum and oral cavity.

**Figure 3:**
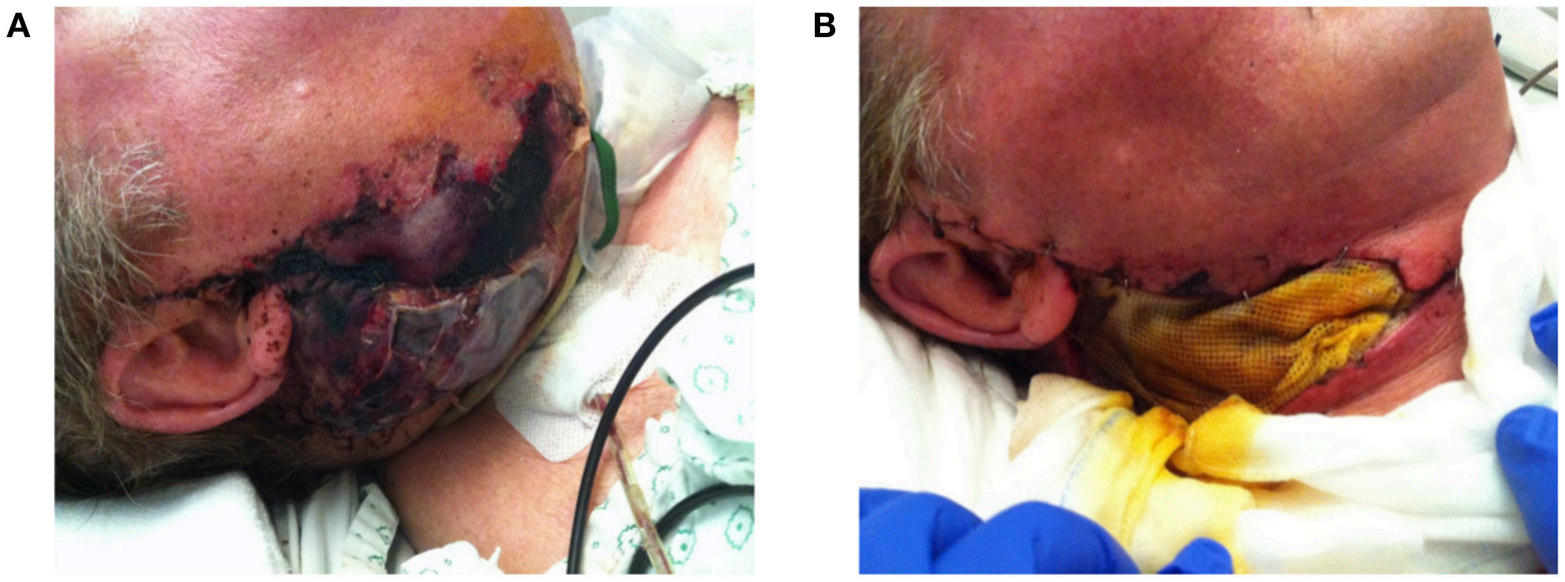
**(A)** Progressive necrosis of a double free flap (fibula + anterolateral femur flap) due to a combination of factors, leading to complete ischemia and death of the free flap. **(B)** The necrotic flap was removed and temporally replaced with a wick.

**Figure 4:**
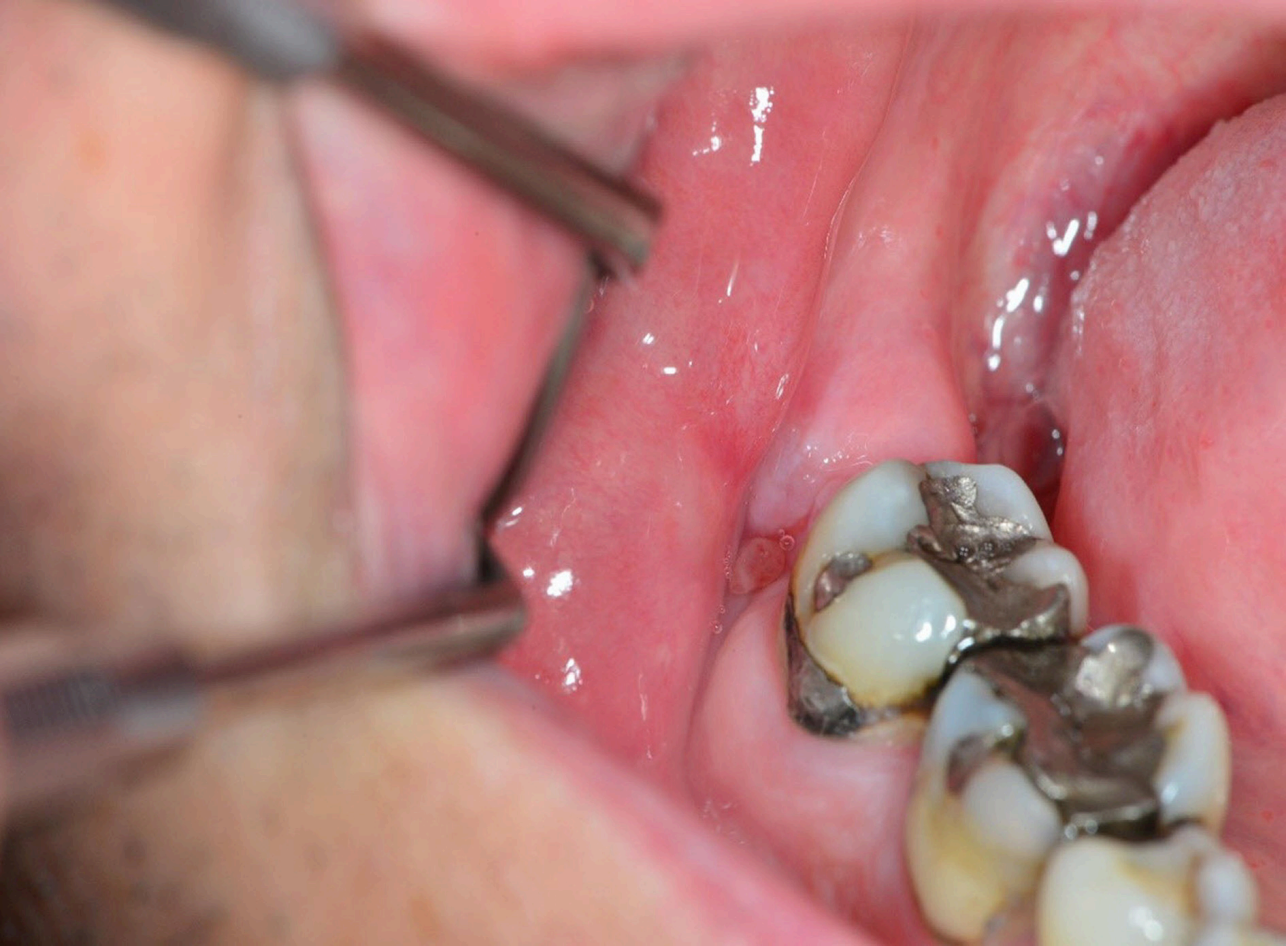
**A poorly healing wound after extraction of 48**. Eight weeks after extraction, the patient continued to complain of pain, and granulation tissue was found in the wound. Wound exploration revealed a pathological fracture together with necrotic bone tissue and bone sequesters.

**Figure 5:**
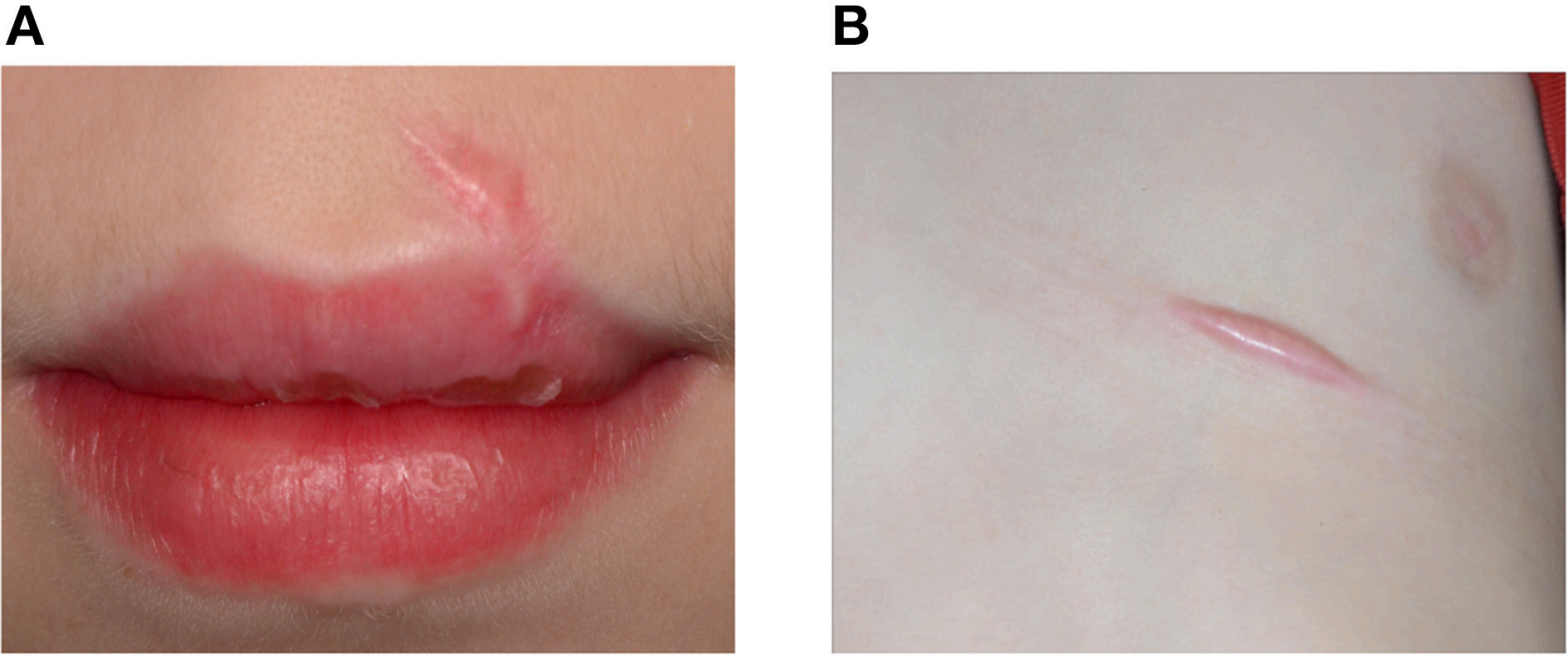
**An 11-year-old boy presented with keloid formation on the upper lip (A) and the thorax (B) after a traffic accident with bicycle**. Hypertrophic scarring and keloid formation indicate abnormal wound healing.

Disturbed wound healing has many manifestations, with the potential for wound healing disturbance at every phase. Clinical manifestations can include excessive bleeding or absence of blood clot formation as seen in alveolitis sicca. Other manifestations can include the granuloma formation (Figures 1A–D), sinus polyps (Figures 2A–C), fistulas, wound dehiscence, ulcers, perforations, wound necrosis, flap necrosis (Figure 3), pus formation, chronic infections with or without granulation tissue formation (Figure 4), keloid formation (Figures 5A,B), fibrosis, and trismus (Adell et al., 1987; Guo and Dipietro, 2010; Glim et al., 2013; Roy et al., 2013; Karamanos et al., 2015). Hom et al. (2009) states that the following clinical signs indicate poor wound healing: persistent inflammation for longer than 7 days, malodorous wound, increased exudate, delayed epithelization, maceration of the surrounding skin, wound dehiscence, and necrotic tissue. A traumatic eosinophil granuloma of the tongue can also be considered a manifestation of poor healing progress. Pro-inflammatory mediators continue to dominate during chronic infections (Hunt et al., 2000).

Cases of nerve damage in the trigeminus area exhibit a special form of tissue damage due to disturbed wound healing. Damage to the branches of the nervus trigeminus due to nerve crushing or pressure can cause dysfunction in the form of neuropathic pain with or without hypoesthesia of the innervation area (Politis et al., 2014). Although, the mechanisms have not yet been elucidated, it is hypothesized that this effect occurs when the nerve and its surrounding environment (vasa nervosum, bone, and adipose tissue) do not sufficiently recover during wound healing.

A typical form of disturbed wound healing involves the root resorption of an element following tooth trauma, tooth transplantation, or reimplantation of an element. Severe damage to the periodontal ligament and cementum transcends the healing capacity, giving rise to tooth ankyloses and replacement resorption (Goldberg, 2011; Dimitrova-Nakov et al., 2014).

## Local factors influencing wound healing in the mouth

It is clinically useful to be aware of the local and general factors that are associated with disturbances of normal wound healing processes (Table 1).

**Table 1:** **Local and general factors that contribute to disturbed wound healing**.

**Local factors**	**General factors**
– Wound size	– Hereditary defects of wound healing
– Wound localization	– Nutritional deficiency
– Postoperative bleeding	– HIV
– Thermal damage	– Cancer
– Perforation to the sinus maxillary	– Old age
– Sharp bone edges	– Diabetes
– Local anesthesia	– Jaundice
– Infection	– Alcoholism
– Hypo-perfusion	– Uremia
– Ischemia	– Immunosuppressives
– Foreign body	– Corticosteroids
– Smoking	– Chemotherapeutics
– Venous insufficiency	– Antiresorptive medication
– Mechanical trauma	– Other medication
– Local toxins	– Vitamin A
– Head or neck irradiation	– Hypothyroidism
– Cancer of the mouth	– Hyperbaric oxygen
– Presence of necrotic tissue	– Anemia
– Local stem cell injections	
– Underlying pathological fractures	
– Injudicious flap design in surgeries	
– Edema	
– Pathological mobility	
– Tooth in the line of a jaw fracture	
– Traumatic occlusion	

### Postoperative bleeding

**Figure 6:**
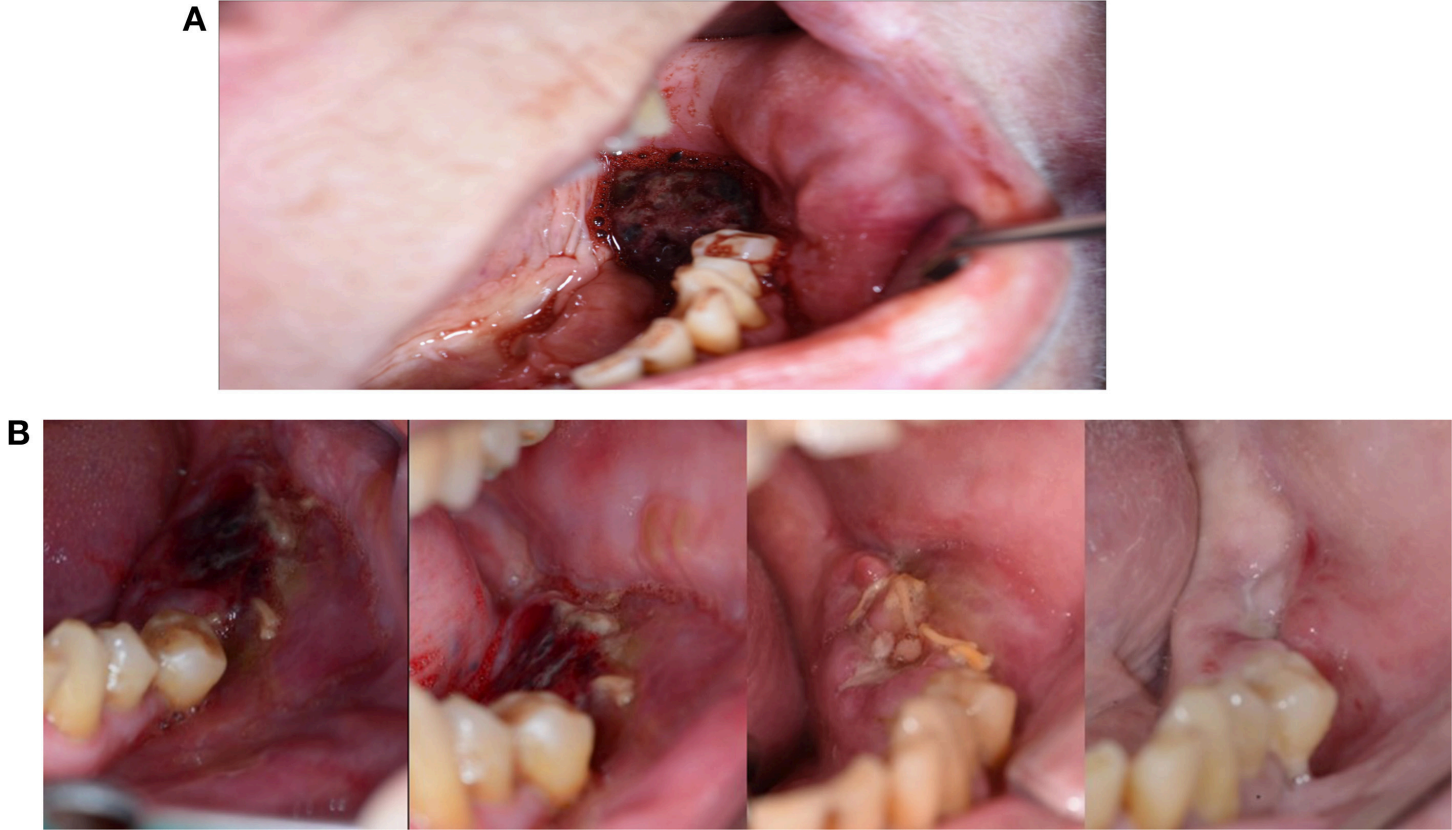
**(A)** A 62-year-old woman presented with repeated postoperative bleeding after extraction of 36. The pictures shows a coagulum. The patient had a history of Crohn's disease for which she took infliximab, and a history of atrial fibrillation for which she used anticoagulants. She also took flecainide and bisoprolol fumarate due to arrhythmias. **(B)** The wound heal after 6 weeks. Wound healing was likely delayed due to the postoperative bleeding, but possibly also due to the use of Infliximab, which is a TNF-α inhibitor.

Postoperative bleeding disturbs granulation tissue formation and slow the healing process (Larjava, 2012). Hereditary and acquired bleeding tendencies only give rise to disturbed wound healing in the presence of pathological bleeding (Rodriguez-Merchan, 2012; Figures 6A,B).

### Hypoxia and oxygen tension

Wounds must have a minimum oxygen tension of 30 mmHg for normal cell division, and a minimum of 15 mmHg for fibroblast proliferation (Schreml et al., 2010). Bacterial destruction by phagocytosis relies on a high partial oxygen pressure in the tissues. Sufficient oxygenation is also required for cell proliferation, angiogenesis, collagen synthesis and re-epithelization. The role of oxygen has mainly been investigated through *in vitro* or animal experiments, and there is not yet sufficient *in vivo* data from humans to truly understand the role of oxygen in wound healing (Yip, 2015). However, it is clear that hypoxia is associated with disturbed wound healing and with bacterial colonization in chronic wounds (Schreml et al., 2010).

### Ischemia

Following dental trauma (avulsion, extrusive luxation, or lateral luxation) or tooth transplantation, a special form of local ischemia can arise in which neovascularization occurs through an open apex, and is followed by osteodentin deposition with a small central pulpal channel that contains a blood supply—termed root canal obliteration. This predominantly occurs in teeth with an open apex, within the first year after trauma. The role of odontoblasts in this process remains unclear (Goldberg, 2011).

### Antrum perforation

Antrum perforation can lead to bad wound healing following extraction of a molar or premolar in the upper jaw.

### Local infection

Infection keeps a wound in an inflammatory state. Such infections are not necessarily prominently visible. A chronic maxillary sinusitis can lead to recurrence of a buccosinusal connection following closure with a Rehrmann flap (Guo and Dipietro, 2010).

### Thermal damage

Excessive monopolar electrocoagulation of the bone, or drilling without cooling, can lead to bone necrosis and the formation of bone sequesters (Guo and Dipietro, 2010; Karamanos et al., 2015).

### Edema

Wound edema restricts the oxygen and nutrient supply to the wound by enlarging the diffusion distance.

### Injudicious flap design in surgeries

**Figure 7:**
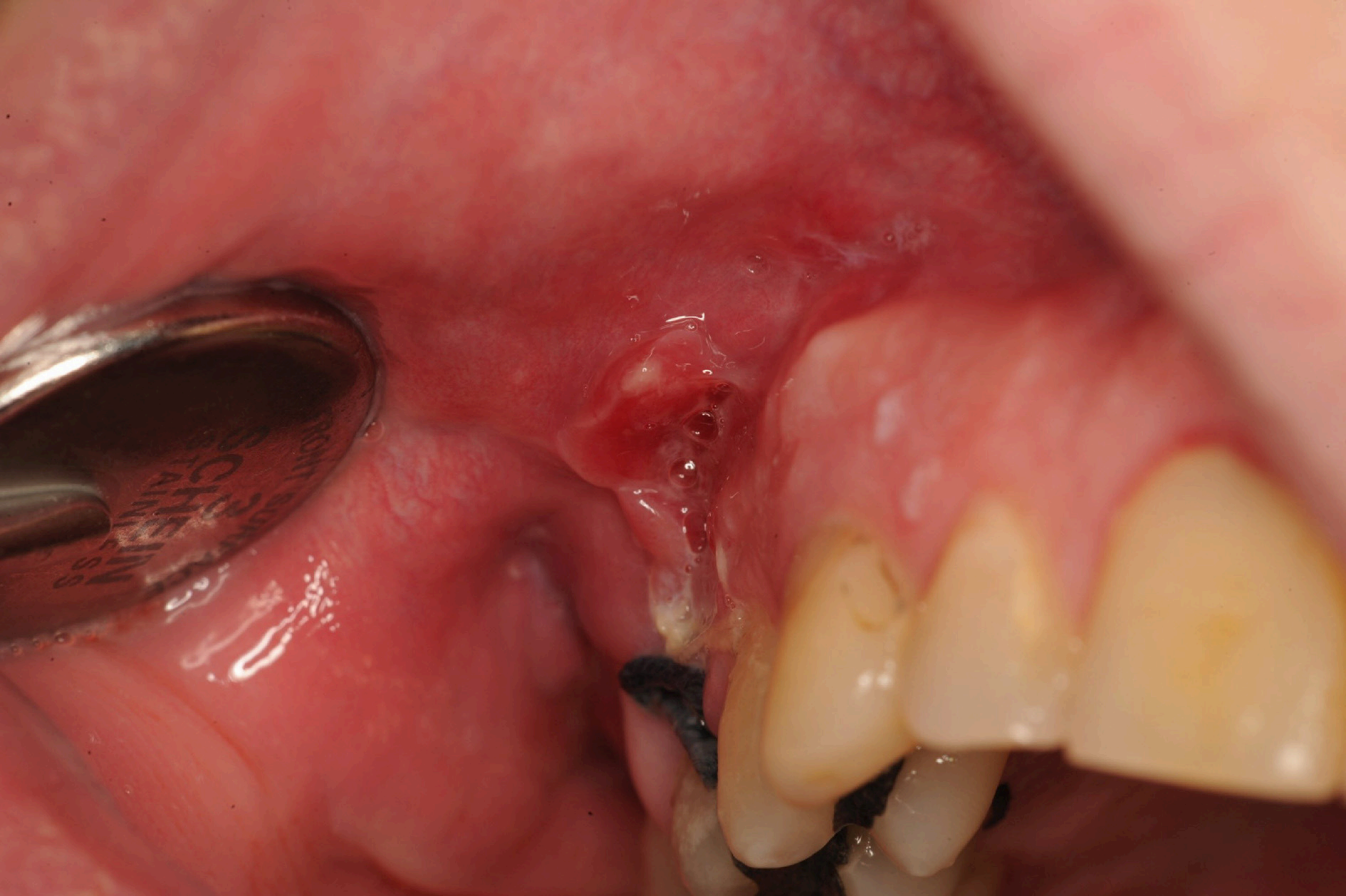
**Buccosinusal connection after apex resection of 16, in which the incision was incorrectly performed on the opening to the sinus rather than on the healthy bone edges**. A broad trapezium-shaped incision would have been desirable.

To avoid flap necrosis and loss of wound covering in periodontal surgery (Lindhe et al., 2015) and in stomatology (Stoelinga et al., 2009), one must follow the basic principles of surgery. This includes ensuring that the flap base is sufficiently wide, limiting the use of monopolar electrocoagulation, ensuring that the wound edges are on healthy bone, and creating no excessive tension on a wound edge. Moreover, Incisions should not be made on an open junction to the maxillary sinus, but rather on healthy bone edges (Figure 7).

### Corpus alienum (foreign body)

**Figure 8:**
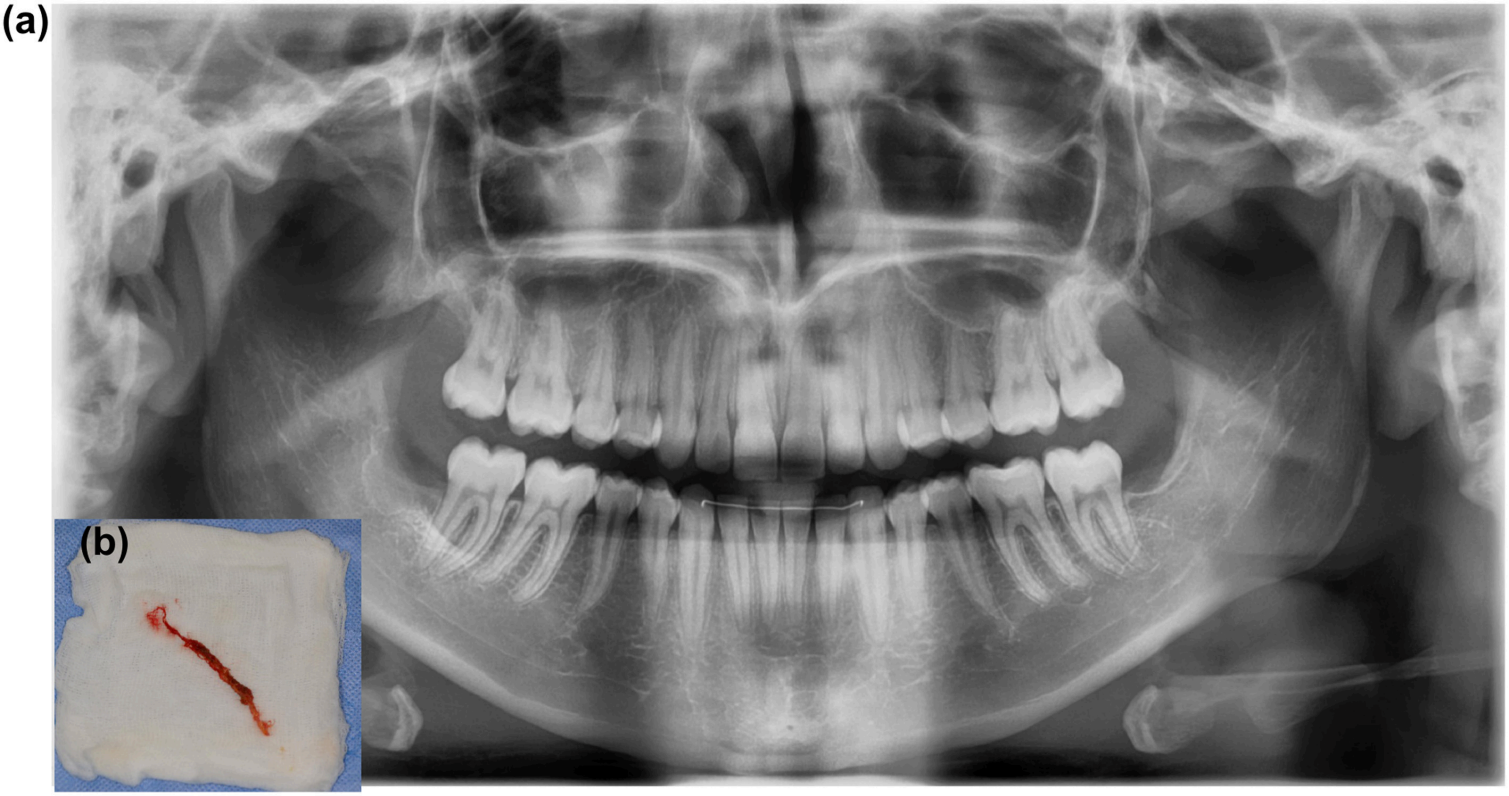
**(A)** A 16-year-old patient was referred due to persisting pain and pus in the extraction cavity of 48. Radiography shows incomplete bone healing in loco 48. **(B)** Wound exploration reveals gauze that remained unnoticed in the wound.

Common corpora aliena in the mouth can vary from a piece of gutta percha or root canal cement to inserted hydroxyapatite granules or osteosynthesis screws. Residual tooth elements, a radix relicta, residual pieces of crown, and bone sequesters can also act as corpora aliena and lead to chronic wound infections. Remaining wicks and compresses can also lead to poor wound healing and latent infections (Figure 8).

Following removal of the wisdom teeth in the mandible, wound healing can be delayed by ischemic necrosis of the buccal edge of the alveolus (Stoelinga et al., 2009). On some occasions, dental implants can act as a corpus alienum and cause severe wound healing problems, such that removal is the only option. Although, a corpus alienum is often visible on radiography, this is not always the case—amalgam tattoos, crown cement around an abutment, and small residues of broken reamers and files can be easily overlooked and cause chronic problems.

### Pathological mobility

Following a Le Fort I fracture or a Le Fort I osteotomy, a pseudo arthrosis can develop due to either bruxism or insufficient rigid fixation. This is also possible in the mandible after a mandibular fracture or osteotomy of the mandible (Adell et al., 1987).

### Tooth element in the line of a jaw fracture

A non-union can be caused by a tooth element in the line of a mandibular fracture, especially when the tooth exhibits a root fracture.

### Traumatic occlusion

**Figure 9:**
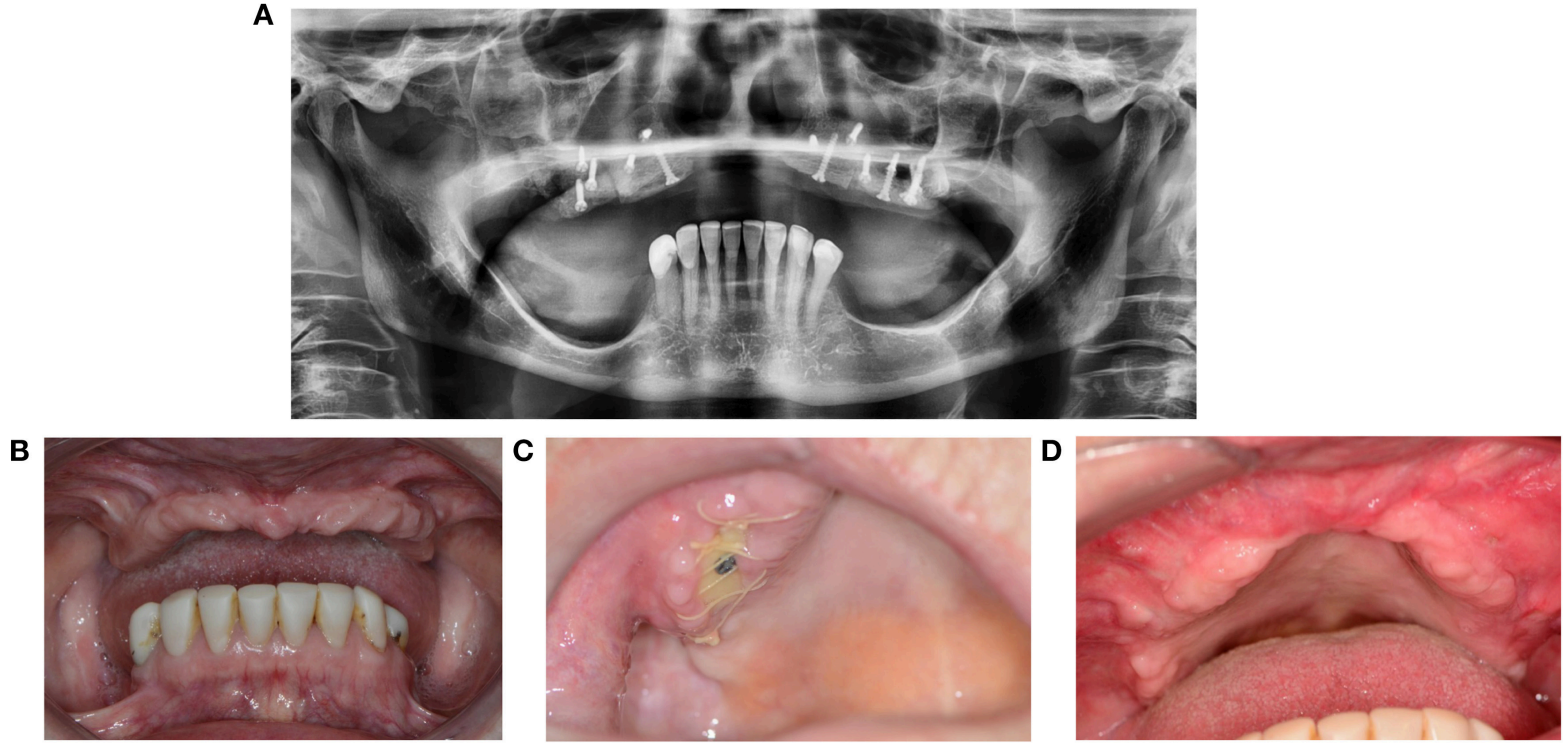
**(A)** 67-year-old patient presented with Kelly syndrome, maxilla atrophy, 20-year history of smoking, and 9-year use of corticosteroids and methotrexate. Maxilla reconstruction was performed through cranial bone. **(B)** Status before reconstruction. **(C)** Wound dehiscence with exposed bone. **(D)** Healing by second intention after removal of the necrotic bone fragment and wound debris.

As opposed to in a toothless maxilla, the presence of natural dentition can threaten a reconstruction when the elements bite on the thin mucosa. Exposure of a bone fragment shortly after reconstruction can threaten the integration (Figures 9A–D). A patient will often present not one but multiple simultaneous contributory factors. Clinical situations that carry higher risk include Kelly syndrome and an egression of the lateral parts of the maxilla with an edentate antagonistic jawbone.

### Sutures

Intraoral wounds are usually sutured with resorbable suture. With regards to resorbable sutures, the tissue reactivity ranges from polydioxanone (PDS) and polyglyconate (Maxon) being the least reactive, to polyglycolic acid (PGA) and polyglactin acid (Vicryl) showing mid-level reactivity, to chromic catgut as the most reactive with tissues (Hollander and Singer, 1999).

### Local anesthesia

In cell cultures and laboratory animals, local anesthetics exhibit an inhibitory effect on wound healing, which is mainly reflected in the inflammatory and proliferation phase of wound healing (Brower and Johnson, 2003). The use of epinephrine underlines this effect (Dogan et al., 2003). Significant clinical effects have not been proven in humans. However, it is recommended to not use large volumes of local anesthetics (tumescent infiltration) in close proximity to surgical incisions in the gingiva.

## General factors influencing the wound healing in the mouth

### Age

Although, age has been proposed to be a cause of bad wound healing, this is not supported by evidence (Karamanos et al., 2015).

### Obesity

Obesity is considered to be a general factor contributing to disturbed wound healing (Guo and Dipietro, 2010). However, the literature does not include data supporting this association with regards to oral wounds.

### Hereditary factors

In the literature, Ehlers-Danlos syndrome and Marfan syndrome are stated to be associated with bad wound healing (Cohen et al., 1992). However, at our tertiary center, we have not noted any problems with wound healing in the mouth among these patients over the last 30 years. Wound healing in the mouth is problematic among patients with osteogenesis imperfecta or epidermolysis bullosa.

### Vitamin A

Macrophage numbers increase with vitamin A intake. A lack of macrophages leads to reduced collagen synthesis and inhibits wound healing.

### Corticosteroids

**Figure 10:**
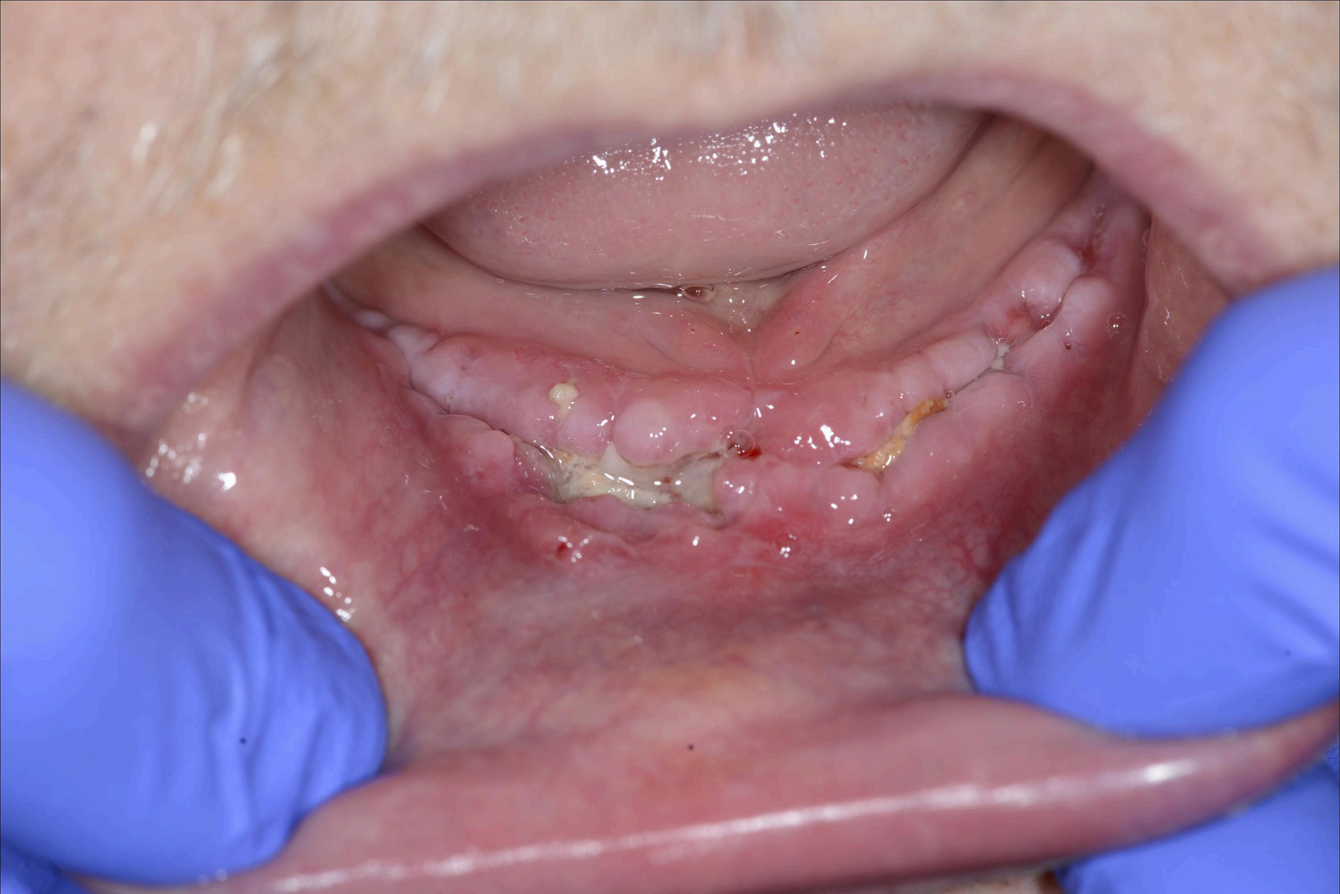
**A 59-year-old male presented with wound healing problems 2 months after placement of four implants in the mandibula**. The patient had been using systemic corticosteroids for the past 15 years due to lung sarcoidosis, and had stopped smoking years ago. Despite excessive alcohol use, neuropathy based on thiamin deficiency, and abnormal liver tests, the patient was able to maintain professional activity and a good social integration.

Corticosteroids have an inhibitory effect on macrophages, leading to a decline in collagen synthesis. Acute administration of high doses of corticosteroids should not impair wound healing, as opposed to chronic corticosteroid administration (Wang et al., 2013; Figure 10).

### Nutrition

Several epidemiological studies report that conditions related to chronic inflammation can be improved by a diet rich in bioactive fat mediators—as present in fish oil, e.g., poly unsaturated fatty acids, eicosapentaenoic acid and docosahexaenoic acid—together with low-dose aspirin (McDaniel et al., 2011). A diet rich in omega-3 fatty acids can be recommended for treatment of chronic inflammatory processes (Simopoulos, 2011; Roy et al., 2013).

**Figure 11:**
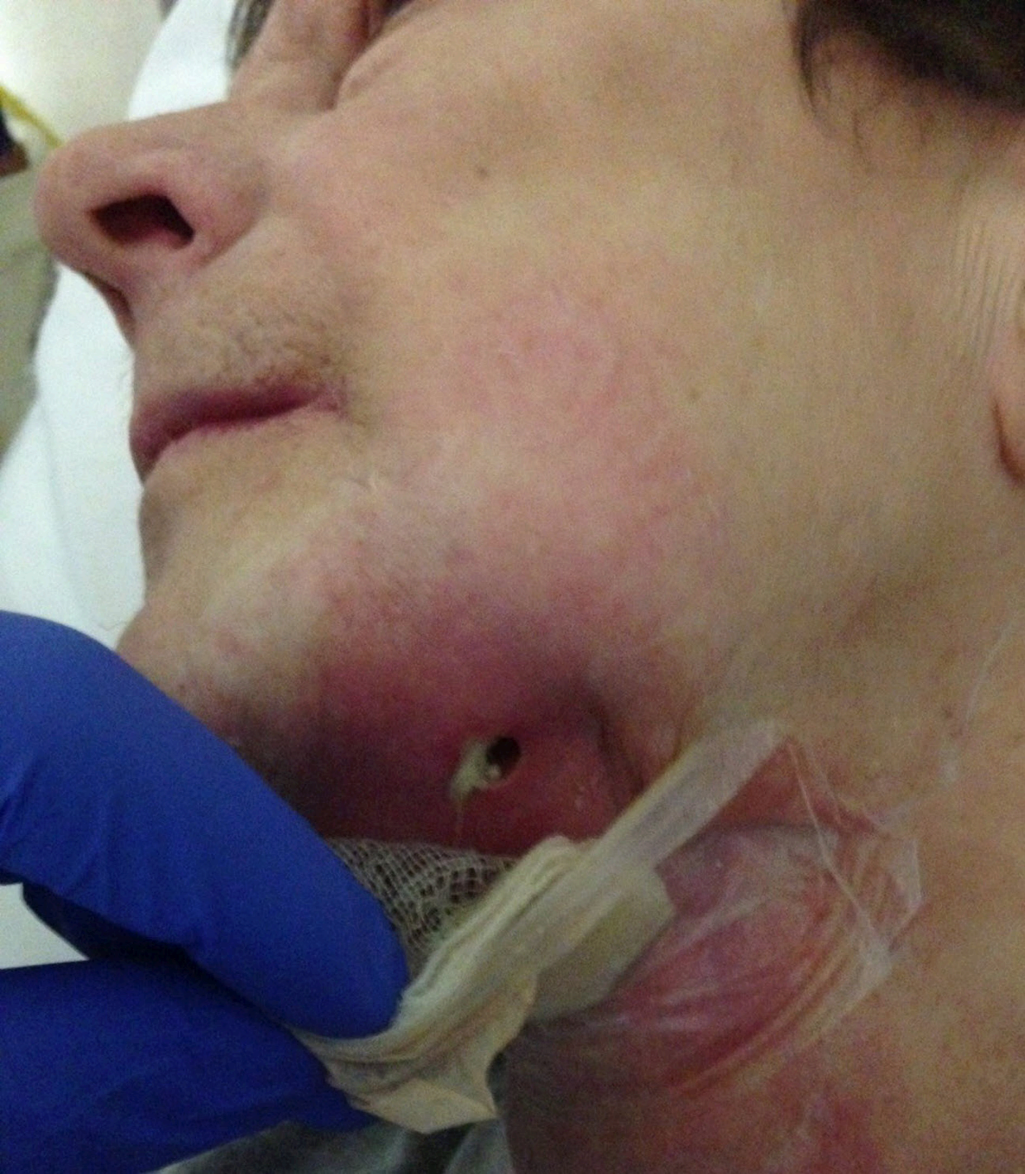
**A 58-year-old female presented with chronic infection, with pus and fistula formation in the neck**. The patient had a history of mouth floor carcinoma, alcoholism, depression, axonal polyneuropathy, Crohn's disease, and nephrotic syndrome. Inadequate oral intake due to pain, with secondary hypoalbuminemia and ion disorders, contributed to poor wound healing and low resistance to infections.

Undernutrition is a problem in wound healing, as is established in daily practice among some patients with mouth cancer combined with ethanol abuse (Figure 11), and in depressed elderly patients who are socially isolated. Protein and vitamin deficiencies particularly influence wound healing (Shafer et al., 1983).

### Diabetes mellitus

**Figure 12:**
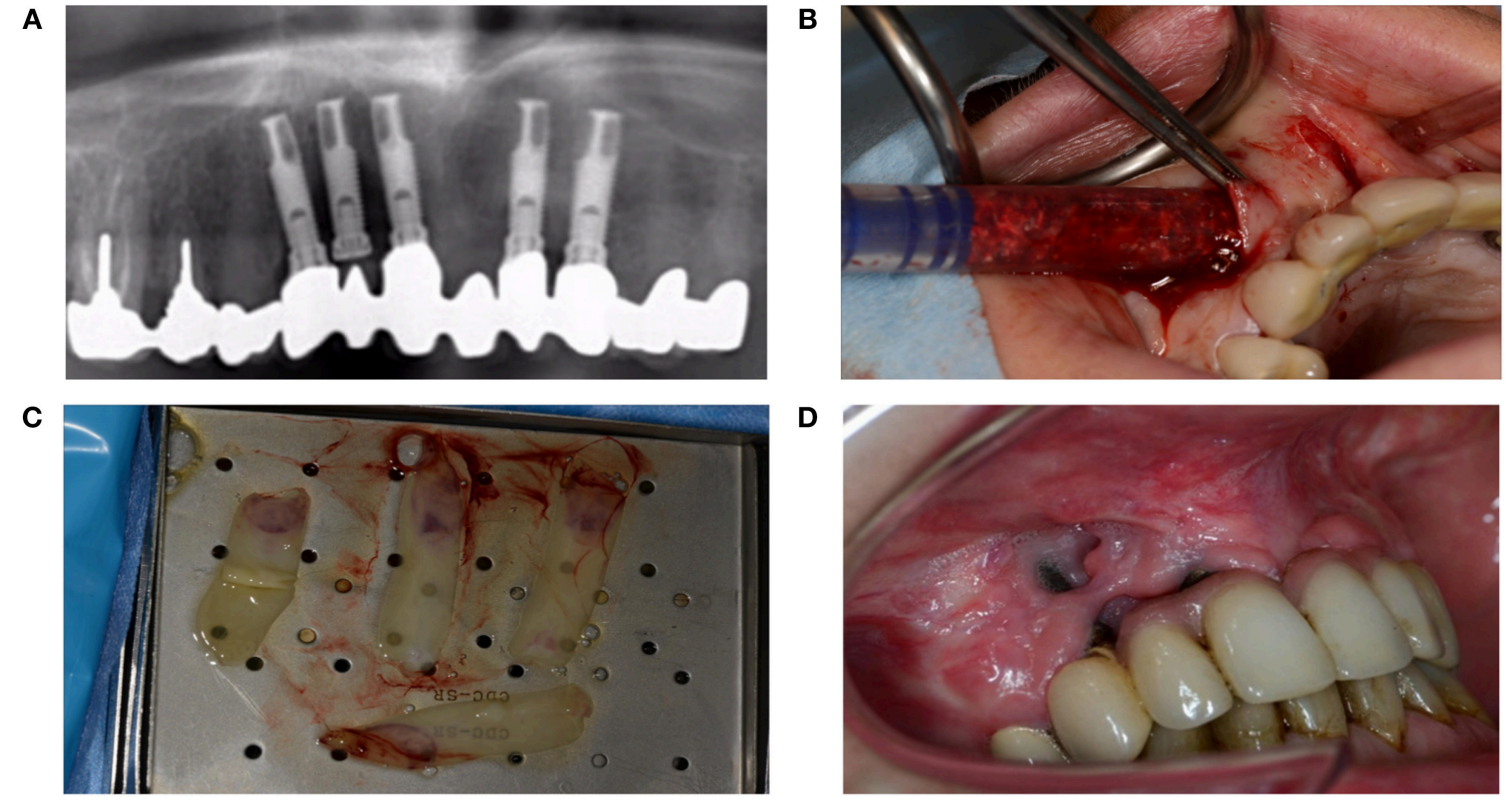
**(A)** A 51-year-old woman presented with peri-implantitis. She was a smoker with unstable diabetes mellitus. Her tooth implants (Nobel Biocare Mk III Ti Unite) had been placed too close to each other, and the central implant in loco 12 had not been used. **(B)** An attempt was made to remove the infectious tissue without removing the implant, and to substitute through artificial bone (BioOss®) and L-PFR membranes. **(C)** LPRF membranes **(D)** End result after recovery attempt, which did not account for numerous factors that impede wound healing.

Diabetes mellitus can severely disturb wound healing, likely due to toxic sorbitol accumulation in tissues, pericapillary albumin deposition that hampers nutrient and oxygen diffusion, and disturbed collagen synthesis and collagen maturation (Broughton et al., 2006). Patients with diabetes also exhibit macrophage dysfunction that causes the inflammatory phase to last longer (Roy et al., 2013; Figures 12A–D).

### Ethanol abuse

**Figure 13:**
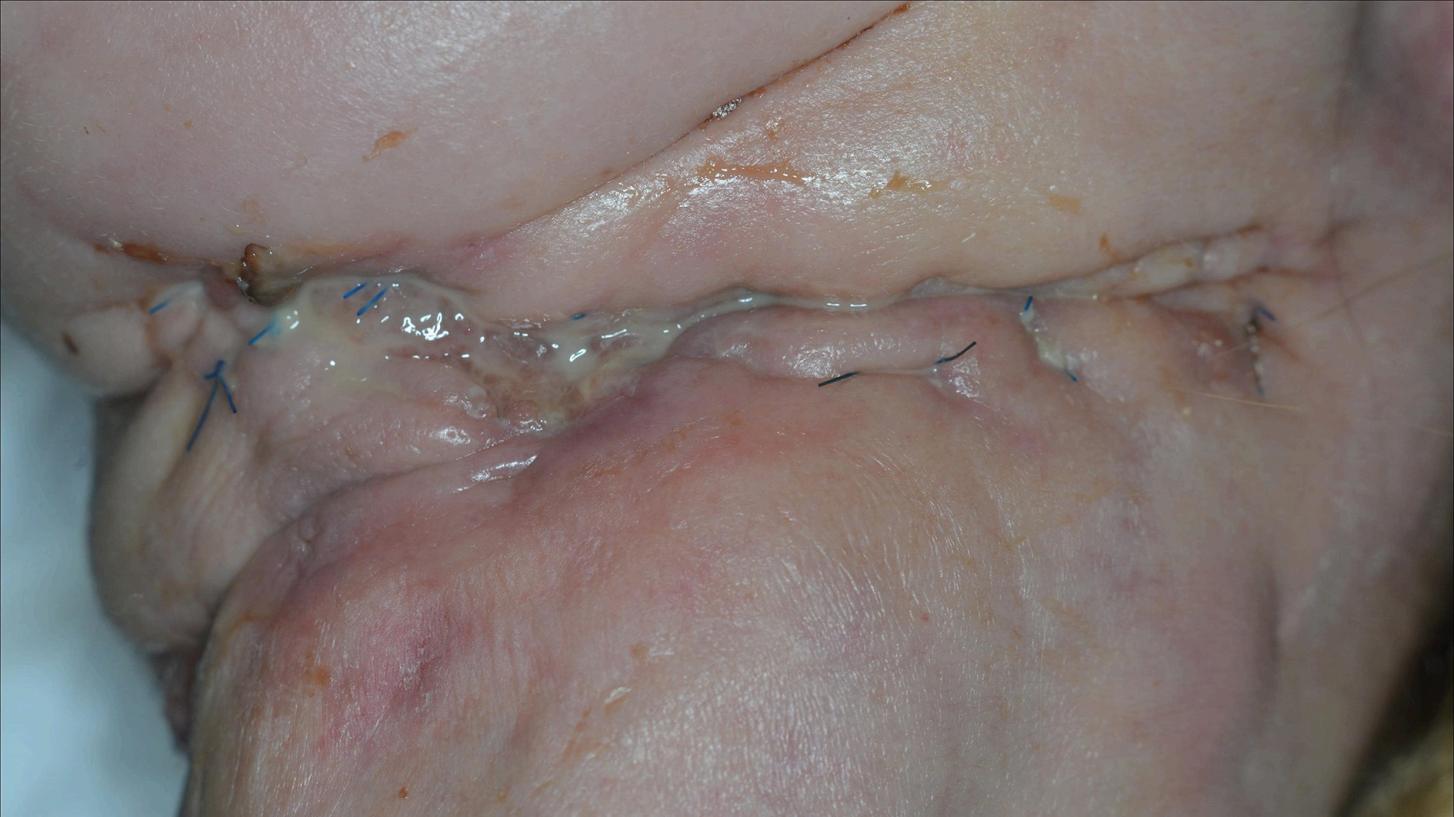
**A 50-year-old patient presented with a neck dissection at the left side**. She was known to abuse ethanol, to suffer from undernutrition, and to have a T4N0M0 spinocellular carcinoma of the left alveolar processes. She was referred due to wound healing problems and recurrent necrosis of the reconstruction through free flaps. Both a fibula flap and a lateral radialis flap had failed. The wound in the neck exhibits inflammation, pus formation, and maceration of the wound edges.

Alcohol metabolism leads to formation of acetaldehyde, reactive oxygen radicals, and other molecules that damage healthy tissue. Almost all phases of wound healing are adversely affected by ethanol consumption (Jung et al., 2011; Figure 13).

### Smoking

Smoking has severe negative effects on all phases of wound healing (Sorensen, 2012). Quitting smoking 4 weeks before surgery has positive effects on the inflammatory phase, but the proliferation phase remains disturbed. Administration of vitamins C and E can decrease the damage in smokers, particularly related to collagen synthesis (Sorensen, 2012).

### Medication

**Figure 14:**
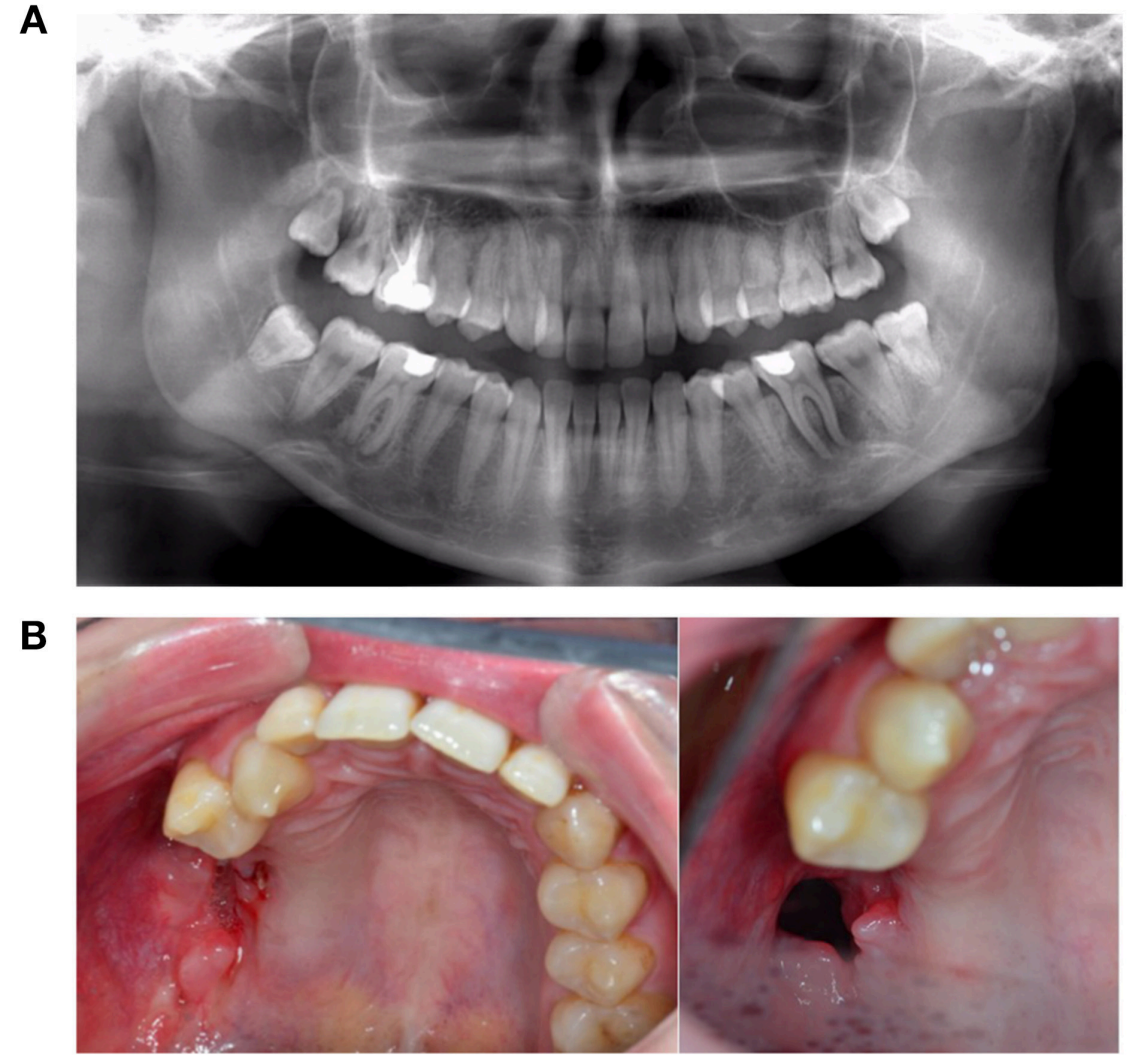
**A 25-year-old female with progressive necrosis of the maxilla, showing loss of all molars in the right maxilla and the alveolar bone, with the emergence of a buccosinusal connection that occurred after ustekinumab use for Crohn's disease treatment**. **(A)** A panoramic radiography from the first consult, where the complaint of loose teeth was formulated. **(B)** Three months after the radiography in panel A, we see the loss of elements 18, 17, 16, and 15. (Photo left). Two months later, an antrum perforation spontaneously occurred.

Bisphosphates, denosumab, and biologicals can cause severe wound healing problems, with clinical manifestations including ulcers, wound dehiscence, bone necrosis, fistulas, and antrum perforations (Figures 14A,B).

### Hyperbaric oxygen

Hyperbaric oxygen therapy is not used for normal wound healing, but is indicated in complex compromised acute injuries with tissue crushing, as well as in necrotic infections, osteomyelitis, chronic ulcers, and late complications of radiotherapy (Dauwe et al., 2014).

### HIV

HIV-positive patients do not seem to have more complications following tooth extraction (Dodson, 1997), but do show more complication after mandibular fracture treatment (Schmidt et al., 1995). The risk for complications significantly increases when the number of CD4 T-lymphocytes is less than 400 per microliter (Dodson, 1997).

### Chemotherapy—immunosuppression

Chemotherapy for mouth cancer can result in oral mucositis during the active treatment phase, and *in vitro* studies indicate disturbed wound healing during chemotherapy. However, disturbed wound healing is not a clinically significant finding after extractions in patients with a history of chemotherapy (Dietrich and Antoniades, 2012). Blood platelets and white blood cells are highly important in wound healing; therefore, wound healing complications are observed during active immunosuppression or chemotherapy (Dunda et al., 2015). However, this disadvantageous effect on wound healing is not permanent.

### Radiotherapy

Patients who undergo radiation therapy due to cancer of the mouth of more than 50 Gy have much greater risk of problems with wound healing after tooth extraction, particularly in the molar region of the mandible (Sulaiman et al., 2003). Extractions in these patients increase the risk of osteoradionecrosis of the jaws when the radiation dose exceeds 50 Gy (Lee et al., 2009; Tsai et al., 2013) more so when other risk factors are present simultaneous (Nabil and Samman, 2012; Nadella et al., 2015).

Preoperative radiotherapy due to oral cancer is associated with higher complication than postoperative radiotherapy (Momeni et al., 2011; Mucke et al., 2012). The effects of radiation on the wound healing after surgery are dose - and fraction dependent and are based primarily on a chronic vascular injury. This also applies to transfer tissue that undergoes postoperative irradiation in the mouth. Jacobsen et al (Jacobsen et al., 2014) recorded an Implant survival rates of 86% in non-irradiated grafted fibular bone, and 38% in irradiated grafted fibular bone.

Fibrosis, atrophy, contraction of oral mucosa, fistula formation, wound dehiscence, skin flap reconstructive failure, non-healing wound and skin necrosis are documented side effects of radiation therapy (Dormand et al., 2005; Gieringer et al., 2011; Haubner et al., 2012).

### Anemia

In maxillo-facial reconstructions using free flaps, low hemoglobin levels should be avoided, especially when the flap exhibits characteristics of ischemia (Xie et al., 2015).

## Conclusion

Wound healing in the mouth occurs in the presence of many challenges, including a high bacterial and viral load, and usually proceeds undisturbed and with preserved oral function. When a facial or intra-oral wound presents a disturbed healing process, it is recommended to conduct a thorough and judicious examination to eliminate or correct underlying local or general factors.

## Author contributions

CP, Initiated the review and assist in writing and review. JA, Write the review. RJ and JS, Reviewed the manuscript.

### Conflict of interest statement

The authors declare that the research was conducted in the absence of any commercial or financial relationships that could be construed as a potential conflict of interest.
